# Segmental choroidal vascularity in Acute Vogt-Koyanagi-Harada disease

**DOI:** 10.1186/s40942-025-00789-9

**Published:** 2026-01-06

**Authors:** Ninan Jacob, Niroj Kumar Sahoo, Jay Chhablani, Abdul Rasheed Mohammed, Kiran Kumar Vupparaboina, Jyothirmai G, Saarang Hansraj, Mudit Tyagi

**Affiliations:** 1 Anant Bajaj Retina Institute, Kode Venkatadri Chowdary Campus, LV Prasad Eye Institute, Vijayawada, Andhra Pradesh India; 2 UPMC Eye Center Eye and Ear Institute, Pittsburgh, PA USA; 3 School of Optometry and Vision Science, University of Waterloo, Waterloo, ON Canada; 4 University of Pittsburgh School of Medicine, Pittsburgh, PA USA; 5 Anant Bajaj Retina Institute, Kallam Anji Reddy Campus, LV Prasad Eye Institute, Hyderabad, Telangana India

**Keywords:** VKH disease, Automated choroidal segmentation, CVI, CSI

## Abstract

**Purpose:**

To analyse the changes in choroidal structures, both in the inner and outer choroid, including choroidal vascularity index (CVI) and choroidal stromal index (CSI) in patients with Vogt-Koyanagi-Harada (VKH) disease during the acute phase and after disease resolution using optical coherence tomography (OCT).

**Methods:**

This was a retrospective, observational study of eyes with acute VKH disease. An automated image binarization protocol was used to analyse the choroid. Choroidal thickness, area, and choroidal vascular and stromal area were calculated using the same protocol. Qualitative changes in the morphology of choroidal vessels were also analysed. Changes in choroidal parameters and correlations before and after treatment were analysed.

**Results:**

We included 55 eyes (29 patients) of acute VKH (9 males and 20 females). Significant reductions were observed in inner, outer, and total choroidal thickness, and in both stromal and vascular areas of the choroid after treatment (*p* < 0.01). However, no significant changes were noted in CVI or CSI. Additionally, 91% of eyes exhibited a horizontally oval or “slit-like” configuration of choroidal vessels following disease resolution, which was associated with a thinner choroid. On regression analysis, worse visual acuity at baseline and higher inner choroidal thickness were associated with greater improvement in visual acuity, and thinner total choroidal thickness at baseline was associated with thinner choroidal thickness at the final visit.

**Conclusion:**

Choroidal structural parameters in both the inner and outer choroid significantly decrease after VKH disease resolution. However, both CVI and CSI remained unchanged. Also, horizontally oval or “slit-like” choroidal vessels may be a novel OCT biomarker of resolved VKH disease.

**Clinical trial registration number:**

N/A

## Introduction

Vogt-Koyanagi-Harada (VKH) disease is described as an autoimmune, multisystem inflammatory disorder, predominantly affecting melanin-containing cells. It results in ocular, neurologic, otologic and dermatological manifestations in genetically susceptible individuals [1–4]. It more commonly affects the adult population, showing a female preponderance [5]. The most frequently affected organ is the eye, which manifests as granulomatous posterior uveitis or panuveitis [6]. The disease presents as diffuse choroiditis, vitritis, exudative retinal detachment, sub-retinal precipitates and papillitis. Acute VKH disease is always bilateral, with unilateral presentation of the disease being a rare occurrence [7]. On histopathological analysis, it is characterised by infiltration of the choroid by lymphocytes, epithelioid cells, histiocytes and multinucleate giant cells [8]. In the acute phase of treatment, high-dose corticosteroids are used; additional immunomodulatory therapy is frequently needed and tapered off slowly [6]. 

The choroid, being the primary site of inflammation in VKH disease, has been assessed by researchers using enhanced depth imaging protocol of spectral domain optical coherence tomography and swept-source OCT, both during the acute phase and following disease resolution [9–11]. The choroidal vascularity index (CVI) is a new metric for the quantitative assessment of vascularity of the choroid that is derived from OCT images. It is the percentage representation of the vascular area relative to the total choroidal area. Agrawal et al. first proposed the use of CVI to monitor the progression of VKH disease [12]. Subsequently, few studies have utilised this new parameter in VKH disease, but have shown conflicting results [12–15]. The acute phase of VKH usually presents with an increased choroidal thickness and an increased CVI value. However, other choroidal conditions like central serous chorioretinopathy have also been described to present with increased CT and CVI. Because both conditions can present with subretinal fluid, it can sometimes be difficult to distinguish between the two entities with the help of OCT, especially in atypical or partially resolved VKH. Furthermore, it is well known that CSCR predominantly causes larger choroidal vessel dilatation with inner choroidal attenuation, while hypothetically, VKH should affect the entire choroid. Thus, our study aimed to assess the choroid, inner and outer choroid separately, using CVI, and to look for indirect biomarkers of activity and resolution.

## Materials and methods

An observational, retrospective study was conducted. The study ran from January 2016 through December 2021. The Institute Ethics Committee granted ethical clearance for the study, and the study followed the principles of the Declaration of Helsinki. Consecutive patients of acute VKH (first episode), were included. The updated VKH diagnostic standards set by the International Committee were used to diagnose every case [8]. The study excluded patients with co-existing retinopathy, other causes of reduced vision, and eyes with inadequate documentation, poor image quality, or where the sclero-choroidal junction was not visible. Data such as age, sex, best corrected visual acuity (BCVA), duration of symptoms before presentation, and duration of disease were obtained through a review of electronic medical records. OCT images were captured by a single trained optometrist using the Triton DRI OCT (Topcon, Tokyo, Japan). Image registration was completed for every follow-up imaging. Analysis was done on a 12 mm horizontal scan across the foveal centre, both at the beginning and after the disease had resolved.

### Definition of choroidal layers

The inner choroidal layer was defined as consisting of both the choriocapillaris and Sattler’s layer, while the outer choroidal layer consisted of the Haller’s layer. The Sattler’s layer and Haller’s layer choroidal vessels were differentiated based on the size and location of the vessels (Sattler’s layer consisting of medium sized and Haller’s layer consisting of large choroidal vessels, and lie in the outer choroid) [16] and the dense stroma rich in intervascular tissue and melanocytes seen in Sattler’s layer, causing a higher degree of light scattering on OCT.

### Choroidal thickness measurement

The sub-foveal region was used to measure the inner and total choroidal thickness. From the outer border of the retinal pigment epithelium (RPE) to the inner border of the Haller layer vessels, the inner choroidal thickness (ICT) was measured. From the outer edge of the RPE to the inner edge of the sclera, the total choroidal thickness (TCT) was measured. The ICT was subtracted from the TCT to determine the outer choroidal thickness.

### Choroidal area measurement and image binarization

**Fig. 1:**
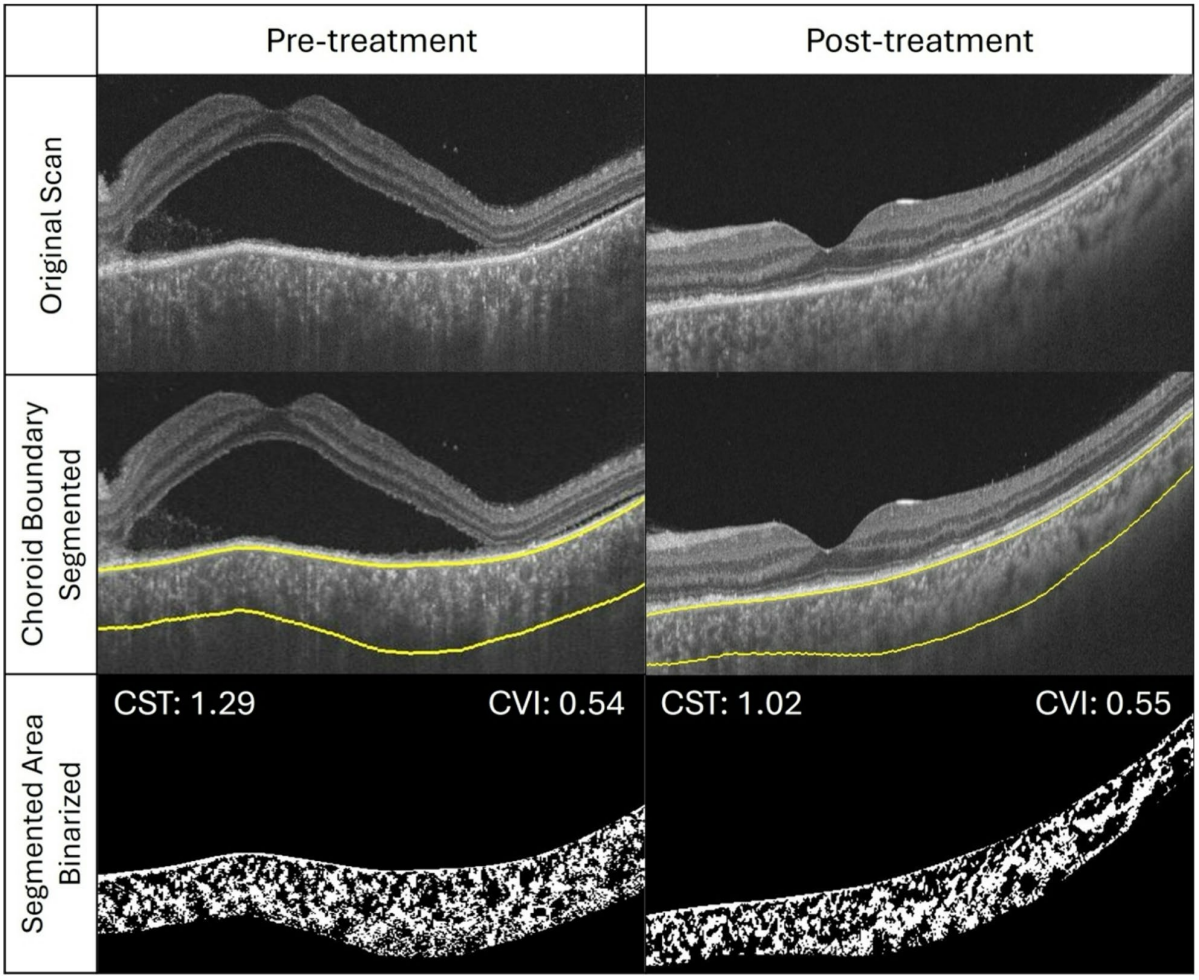
Schematic diagram depicting image binarization process pre and post treatment

**Fig. 2:**
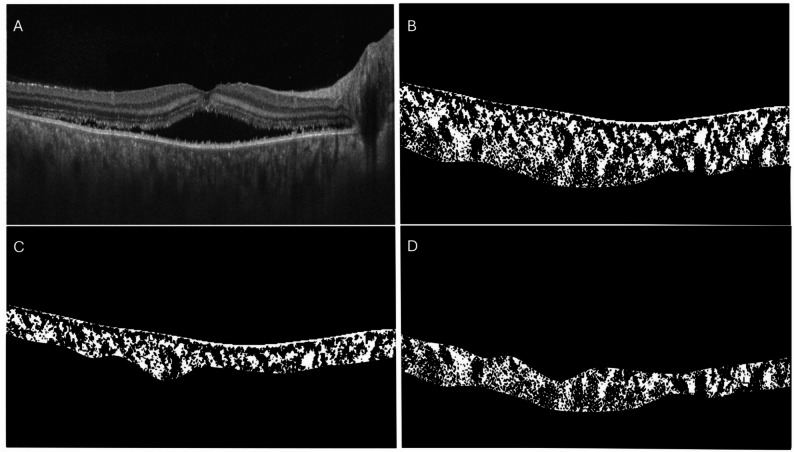
Horizontal swept-source optical coherence tomography scan (6 mm) of a patient presenting with acute VKH disease (**A**); Automated binarized choroidal image of the same patient (**B**); Manual segmentation into inner choroid (**C**) and outer choroid (**D**)

The choroid was analysed using a previously validated algorithm, with good reproducibility (Figs. 1 and 2) [17, 18]. The total horizontal length of the OCT scan was included for image analysis. The algorithm automatically detected the inner and outer choroidal borders, with manual adjustments to the choroidal border performed when necessary. The presumed junction between the inner and outer choroidal layers was manually segmented. The algorithm then divided the choroidal area into dark and bright regions, providing the “dark” choroidal vessel luminal and choroidal areas. The choroidal vascularity index was calculated by dividing the luminal (dark) area by the total choroidal area. The stromal area was then obtained by subtracting the luminal area from the total choroidal area. The choroidal stromal index was calculated by dividing the stromal area by the total choroidal area. These measurements were performed for the inner choroid (choriocapillaris and Sattler layer), outer choroid (Haller layer), and overall choroidal area. The Haller layer was manually segmented by drawing a line passing over the inner border of individual Haller layer vessels. An average of three measurements of each parameter was done to minimise error. We also assessed the presence of choroidal hyper-reflective dots on the OCT scan, defined as hyper-reflective spots with equal or higher reflectivity than the RPE. Additionally, the morphology of the choroidal vessels was observed following disease resolution.

SPSS (SPSS v20.0, IBM Corp., Armonk, NY, USA) and Microsoft Excel (Microsoft Office 2019, Redmond, Washington, United States) were used for the statistical analysis. We based our sample-size estimation based on the study by Agrawal et al. [12] The authors reported that CVI decreased from 70.03 ± 1.93% to 66.94 ± 1.82%. This corresponds to a standardized effect size of approximately 1.6. Using a two-sided α of 0.05 and 90% power would require ~ 10 eyes in a paired longitudinal design. Our cohort included of 55 eyes from 29 consecutive acute VKH patients with good quality image, (therefore providing ample statistical power) to minimise selection bias. Data normality was assessed using the Kolmogorov-Smirnov test. Snellen visual acuity was converted into logMAR units. For normally distributed data, the variables were analysed before and after treatment using a two-tailed paired T-test; for non-normally distributed data, the Wilcoxon Signed rank was used. The relationship between different parameters was examined using a Pearson correlation test, and predictive analysis was done using the linear regression function of generalised estimated equations, taking into consideration fellow eye status. Variables having p-value < 0.05 in the univariable analysis, were added to the multivariable model. A p-value of less than 0.05 was considered statistically significant.

## Results

A similar cohort of patients as in our previous paper was included in the study [19]. The study comprised 55 eyes of 29 acute VKH patients, 9 males and 20 females. Three eyes from three patients were excluded due to poor image quality or poor documentation. The age ranged from 9 to 57 years, with an average of 33.76 ± 12.12 years. The mean duration of symptoms before presentation was 15.43 +/- 16.75 days, and the mean follow-up time was 89.32 +/- 86.49 days. All patients were treated with systemic corticosteroids, with nearly 34% of patients (10/29) requiring baseline intravenous corticosteroids based on the treating physician’s discretion. The baseline characteristics of the patient have been summarised in Table 1.

**Table 1:** Baseline characteristics of the patients

Baseline demographic data	
Mean age (Years)	33.76+/- 12.12
Gender	Males 9 (31.03%) Females 20 (68.96%)
Mean duration of symptoms (Days)	15.43+/-16.75
Eyes involved	55
Patients who received IVMP at baseline	10 (34.48%)

### Differences and correlations of choroidal indices

**Fig. 3:**
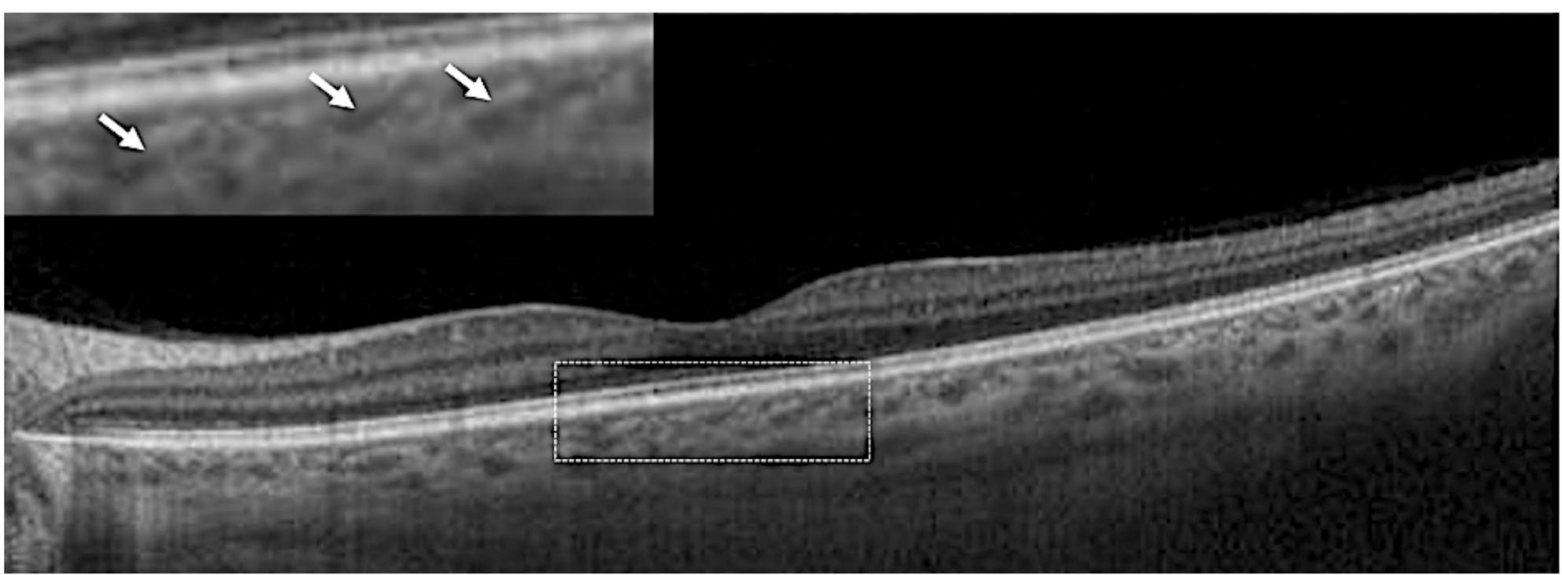
Horizontal swept-source optical coherence tomography scan (6 mm) showing a slit-like architecture of choroidal vessels (*arrows in the inset*) following resolution of the disease

A summary of various choroidal parameters at baseline and at follow-up is shown in Table 2. The best corrected visual acuity at baseline was 0.600 ± 0.427 logMAR. The inner choroidal thickness, inner choroidal vascular area and inner choroidal area comprised a mean of 24.1%, 34.1%, and 25.8% of the total choroidal thickness, total vascular area and total choroidal area, respectively. By the end of the follow-up, the cohort’s mean visual acuity had significantly improved from 0.600 ± 0.427 logMAR to 0.103 ± 0.232 (*p* < 0.01). After the treatment, there was a significant reduction in the inner and total choroidal thickness, as well as the inner choroidal stromal and vascular area and the total choroidal stromal and vascular area, according to OCT-based measurements. However, no significant difference in the choroidal stromal or vascularity index was noted following the resolution of the disease in both the inner choroid and total choroid (Table 2). Good correlations were observed between various inner and outer choroidal parameters, both at baseline and after resolution (Table 3). While choroidal hyperreflective dots were noted in 85.4% (47/55) of eyes during the acute stage of the disease on OCT analysis, there was a complete absence of these hyperreflective dots at the end of the treatment. In 91% (50/55) of eyes, choroidal vessels assumed a horizontally oval or “slit-like” configuration following disease resolution (Fig. 3). The final CT measurement in eyes with a “slit-like” configuration (421.6 ± 107.8 microns) was lower than that without this morphology (579.9 ± 87.3 microns) (*p* = 0.01).

**Table 2:** Comparison of parameters before and after complete resolution of subretinal fluid

Parameters	Baseline	Post resolution	Mean percentage change	*p*-value
Inner choroidal thickness (microns)	142.432 ± 41.748	93.453 ± 3 4.845	-34.39	< 0.01
Total choroidal thickness (microns)	589.831 ± 100.622	435.957 ± 115.009	-26.09	< 0.01
Inner choroidal vascular area	9140.581 ± 5146.506	*4292 (3041-11080.5)	-53.04	^#^<0.01
Inner choroidal stromal area	*3686 (2204-12536.5)	*1896 (1630-9688.5)	-48.56	^#^<0.01
Total choroidal vascular area	26799.309 ± 8430.731	19181.909 ± 9558.372	-28.42	< 0.01
Total choroidal stromal area	*12,848 (9534–36203)	*6512 (4470.5-32947.5)	-49.32	^#^<0.01
Inner choroidal vascularity index	0.618 ± 0.123	0.608 ± 0.125	-1.62	0.58
Inner choroidal stromal index	0.374 ± 0.126	0.385 ± 0.128	2.94	0.57
Total choroidal vascularity index	0.608 ± 0.112	*0.65 (0.46–0.72)	6.91	^#^0.89
Total choroidal stromal index	*0.35 (0.305–0.525)	0.383 ± 0.140	9.43	^#^0.48

*Median (Interquartile range); # Wilcoxon Signed Rank test

**Table 3:** Correlation between different parameters

Parameters	*r*	*p*-value
Inner choroidal area and outer choroidal area pre treatment	0.785	< 0.01
Inner choroidal area and outer choroidal area post treatment	0.867	< 0.01
Inner choroidal thickness and total choroidal thickness pre-treatment	0.49	< 0.01
Outer choroidal thickness and total choroidal thickness pre-treatment	0.91	< 0.01
Inner choroidal thickness and total choroidal thickness post-treatment	0.3712	< 0.01
Outer choroidal thickness and total choroidal thickness post-treatment	0.9533	< 0.01
Change in inner choroidal thickness and change in total choroidal thickness	0.3724	< 0.01
Change in outer choroidal thickness and change in total choroidal thickness	0.9344	< 0.01

### Influence on final CT and change in BCVA

On analysing the factors that were associated with change in BCVA (Table 4), baseline BCVA and inner choroidal thickness were found to be significant in univariate analysis. On multivariate analysis, baseline BCVA (*p* < 0.01) and baseline inner choroidal thickness (*p* = 0.04) were seen to be significant. Similarly, factors associated with choroidal thinning at baseline (Table 5) were total vascular area at baseline (*p* < 0.01), inner choroidal thickness at baseline (*p* < 0.01), total choroidal thickness at baseline (*p* < 0.01), and baseline BCVA (*p* < 0.01). Due to high collinearity between inner and total choroidal thickness, only total choroidal thickness was included in the multivariate analysis. On multivariate analysis, only total choroidal thickness was seen to be significant (*p* = 0.04).

**Table 4:** Factors affecting change in best corrected visual acuity

	Univariable			Multivariable*		
	Regression co-efficient (B)	95% CI of B	*p*-value	Regression co-efficient (B)	95% CI of B	*p*-value
Baseline BCVA	-0.93	-1 to -0.85	< 0.01	-0.9	-0.98 to -0.83	< 0.01
Inner choroidal thickness at baseline	-0.003	-0.006 to -0.001	0.02	-0.001	-0.001 to 0	0.04

BCVA: Best corrected visual acuity

*Variables having *p* < 0.05 were entered into the multivariate model

**Table 5:** Regression: factors affecting final choroidal thickness

	Univariable			Multivariable*		
	Regression co-efficient (B)	95% CI of B	*p*-value	Regression co-efficient (B)	95% CI of B	*p*-value
Total vascular area at baseline	0.004	0 to 0.007	0.03	0.003	-0.001 to 0.006	0.13
Baseline BCVA	72.5	2.3 to 142.7	0.04	35.7	-35.7 to 107.1	0.32
Total choroidal thickness at baseline	0.39	0.11 to 0.66	< 0.01	0.3	0.02 to 0.6	0.04

BCVA: Best corrected visual acuity

*Variables having *p* < 0.05 were entered into the multivariate model

## Discussion

In our study, we compared changes in choroidal parameters, including choroidal thickness, choroidal stromal area, and choroidal vascular area, in both the inner and total choroid, both during the acute stage of VKH and following disease resolution. We observed a significant decrease in these parameters. Additionally, both the inner and total choroid’s CVI and CSI showed no significant change after resolution. It was also seen that age and duration of follow-up are important factors in predicting final visual acuity.

Choroidal inflammation is the primary pathophysiological mechanism of VKH disease. The thickening of the choroid observed in acute VKH can be attributed to both inflammatory cell infiltration and increased exudation [14, 20]. The notable reduction in choroidal thickness after disease resolution, seen across the inner, outer, and total choroid, indicates the widespread involvement of the choroid in VKH disease. Although previous studies have demonstrated that the choroid is thicker during the active stage of VKH compared to the healthy population, with a significant decrease after resolution [11, 13, 21], this study is unique in its detailed analysis of the inner choroid. These findings are further supported by histopathological evidence of diffuse inflammation of the middle and outer layers of the choroid caused by inflammatory cells during acute VKH disease [8]. To improve the analysis of the inner choroid (containing choriocapillaris and Sattler’s layer vessels) and the outer choroid (containing Haller’s layer vessels), we employed an algorithmic approach that delineates Haller’s layer from Sattler’s layer, as previously described in the literature [22, 23]. 

We believe that the choroidal stromal area increases as a result of diffuse infiltration of the choroid by inflammatory cells. Additionally, stromal oedema is caused by the secretion of various inflammatory molecules, including vascular endothelial growth factor and tumour necrosis factor-alpha [14]. On OCT analysis, we noted numerous hyperreflective dots distributed throughout the choroid during the acute stage of the disease, which are suggestive of inflammatory cells. Furthermore, these hyperreflective dots disappear after the disease resolves. This could account for the significant decline in the choroidal stromal area after the disease resolved. A similar finding of decreased CSA post-resolution of the disease was noted by Kawano et al. in their study [14]. In contrast, the study by Jaisankar et al. [13] showed an increase in CSA following the resolution of the disease. However, their finding did not reach statistical significance.

We also noted a significant decrease in the choroidal vascular area following disease resolution. It is hypothesised that endothelial nitric oxide synthase can be upregulated by the cytokines released as a result of choroidal inflammation, causing choroidal vessels to dilate [14, 24, 25]. Additionally, choroidal inflammation can cause stasis of choroidal blood flow, leading to increased dilation of choroidal vessels [12, 13]. This stasis is, however, reversed following systemic treatment with corticosteroids, which has been demonstrated by laser speckle flowgraph [26, 27] and indocyanine green angiography [28]. In an analysis of other papers, while similar results were obtained by Kawano et al. [14], Jaisankar et al. [13] showed no change in the vascular area following disease resolution, but their finding did not reach statistical significance.

Based on correlation analysis, we observed a significant correlation between the inner and outer choroidal areas during the active stage of the disease. Additionally, there was a strong correlation between the total choroidal thickness and the inner and outer choroidal thickness, respectively. Similarly, following disease resolution, there was a significant correlation between the inner and outer choroidal area as well as the inner and outer choroidal thickness with the total choroidal thickness. In the study by Hirooka et al. [27], they noticed that the decrease in outer choroidal thickness correlated with the decrease in total choroidal thickness following the initiation of systemic corticosteroid therapy, but such a correlation could not be seen for the inner choroidal thickness. They concluded that in patients with VKH disease receiving systemic corticosteroids, the choroidal outer layer plays a major role in the recovery from choroidal thickening. However, they did not measure the choroidal thickness during the acute stage of the disease. This is in contrast to our analysis, where we could show a significant correlation between the change in the inner and outer choroidal thickness with the total choroidal thickness. However, in our study too, we saw a more significant correlation with the change in outer choroidal thickness as compared to the change in inner choroidal thickness. Taken together, we believe that while both the inner and outer choroid are affected, the outer choroid is more severely affected than the inner choroid at the start of the disease.

Interestingly, on analysing several factors affecting change in visual acuity, we found that worse baseline BCVA and higher inner choroidal thickness were associated with greater improvement in BCVA. Similarly, a lower total choroidal thickness at baseline was associated with lesser choroidal thickness at the final visit on multivariate analysis, although inner choroidal thickness, baseline BCVA and total choroidal vascular area at baseline were significant on univariate analysis. This emphasises the importance of the total choroidal thickness and the inner choroidal thickness on the final visual recovery and the final choroidal thickness.

In terms of the choroidal stromal index and choroidal vascularity index, we did not achieve a significant difference between the acute stage of the disease and post-resolution. While Agarwal et al. [12] and Jaisankar et al. [13] reported a significant decrease in CVI at the end of their treatment, Kawano et al. [14] showed that CVI significantly increased 1 week after starting steroid treatment. Liu et al. [15] observed in their cohort that while there was a significant drop in CVI during the recurrent attack of anterior uveitis in VKH patients, the CVI increased once the attack resolved. Jaisankar et al. [13] also noted a significant increase in CSI at the end of their treatment. While no definitive answer can be given for the conflicting results in the above-mentioned studies, we believe that CSI and CVI, being a ratio, were not significantly affected, as significant decreases were noted in both the choroidal stromal and vascular area (numerator) and the choroidal thickness (denominator), which helped maintain the overall ratio in our study.

Furthermore, we observed a peculiar pattern in OCT, where, after disease resolution, the choroidal vessels assumed a horizontal oval or slit-like appearance, seen in 91% of eyes in our cohort. Additionally, these changes were observed in eyes with a thinner choroidal thickness at resolution. “Slit-like” vessels referred to markedly flattened, thin vessels with a length-to-height disproportion greater than that of normally seen horizontally oval Haller vessels. We believe that some degree of choroidal remodelling occurs as a result of the inflammatory insult and steroid treatment, which may be responsible for the alteration in vessel architecture. These vessels could represent a collapse of the dilated and congested choroidal vessels that lose their volume upon resolution. However, further validation of this observation is necessary in future studies.

The use of a single OCT operator, a single machine for image capture, a higher sample size compared to previous studies on CVI in VKH, and the utilization of high-quality images during the acute stage and follow-up are among this study’s main strengths. However, the retrospective nature of the study was a limitation. There may have been unavoidable artefacts in the OCT due to the inflammation which may have influenced the analysis. Also, there could have been some degree of error in the detection of the outer choroidal boundary and the boundary between the inner and outer choroid. However, the measurements were repeated and averaged, which would have reduced the error. Finally, although CVI and CSI did not show significant longitudinal change, the true effect size may have been small, and thus our study may have been underpowered to detect subtle stromal–luminal shifts, and a Type II error could not be completely excluded.

## Conclusion

In conclusion, while a significant change can be noted in various choroidal structures both in the inner and total choroid following the resolution of VKH, including the presence of slit-like choroidal vessels, no significant changes were noted in the choroidal stromal and vascularity index. Inner choroidal thickness and total choroidal thickness were seen to be important factors associated with visual recovery and choroidal thinning. We believe that these changes can be particularly beneficial in differentiating VKH from other possible mimickers in atypical presentations. However, future studies with prospective designs and standardised imaging protocols would be beneficial to validate it further.

## Data Availability

The data that support the findings of this study are available from the corresponding author N.K.S. upon reasonable request.

## Declarations

### Presentation at a conference

Nil

## References

[1] Burkholder BM. Vogt-Koyanagi-Harada disease. Curr Opin Ophthalmol. 2015;26(6):506–11. 26448042 10.1097/ICU.0000000000000206

[2] Liu XY, Peng XY, Wang S, You QS, Li YB, Xiao YY, FEATURES OF OPTICAL COHERENCE TOMOGRAPHY FOR THE DIAGNOSIS OF VOGT-KOYANAGI-HARADA DISEASE, et al. Retina Phila Pa. 2016;36(11):2116–23. 10.1097/IAE.0000000000001076 27145255

[3] O’Keefe GAD, Rao NA. Vogt-Koyanagi-Harada disease. Surv Ophthalmol. 2017;62(1):1–25. 27241814 10.1016/j.survophthal.2016.05.002

[4] Sakata VM, da Silva FT, Hirata CE, de Carvalho JF, Yamamoto JH. Diagnosis and classification of Vogt-Koyanagi-Harada disease. Autoimmun Rev. 2014;13(4–5):550–5. 24440284 10.1016/j.autrev.2014.01.023

[5] Du L, Kijlstra A, Yang P. Vogt-Koyanagi-Harada disease: novel insights into pathophysiology, diagnosis and treatment. Prog Retin Eye Res. 2016;52:84–111. 26875727 10.1016/j.preteyeres.2016.02.002

[6] Damico FM, Kiss S, Young LH. Vogt-Koyanagi-Harada disease. Semin Ophthalmol. 2005;20(3):183–90. 16282153 10.1080/08820530500232126

[7] Forster DJ, Green RL, Rao NA. Unilateral manifestation of the Vogt-Koyanagi-Harada syndrome in a 7-year-old child. Am J Ophthalmol. 1991;111(3):380–2. 2000916 10.1016/s0002-9394(14)72334-7

[8] Read RW, Holland GN, Rao NA, Tabbara KF, Ohno S, Arellanes-Garcia L, et al. Revised diagnostic criteria for Vogt-Koyanagi-Harada disease: report of an international committee on nomenclature. Am J Ophthalmol. 2001;131(5):647–52. 11336942 10.1016/s0002-9394(01)00925-4

[9] Spaide RF, Koizumi H, Pozzoni MC. Enhanced depth imaging spectral-domain optical coherence tomography. Am J Ophthalmol. 2008;146(4):496–500. 18639219 10.1016/j.ajo.2008.05.032

[10] Fong AHC, Li KKW, Wong D. Choroidal evaluation using enhanced depth imaging spectral-domain optical coherence tomography in Vogt-Koyanagi-Harada disease. Retina Phila Pa. 2011;31(3):502–9. 10.1097/IAE.0b013e3182083beb 21336069

[11] Nakai K, Gomi F, Ikuno Y, Yasuno Y, Nouchi T, Ohguro N et al. Choroidal observations in Vogt-Koyanagi-Harada disease using high-penetration optical coherence tomography. Graefes arch clin exp ophthalmol Albrecht von Graefes arch Klin exp ophthalmol. 2012 July;250(7):1089–95. 10.1007/s00417-011-1910-7 22240936

[12] Agrawal R, Li LKH, Nakhate V, Khandelwal N, Mahendradas P. Choroidal vascularity index in Vogt-Koyanagi-Harada disease: an EDI-OCT derived tool for monitoring disease progression. Transl Vis Sci Technol. 2016 July;5(4):7. 10.1167/tvst.5.4.7 PMC4970799 27525196

[13] Jaisankar D, Raman R, Sharma HR, Khandelwal N, Bhende M, Agrawal R, et al. Choroidal and retinal anatomical responses following systemic corticosteroid therapy in Vogt-Koyanagi-Harada disease using Swept-Source optical coherence tomography. Ocul Immunol Inflamm. 2019;27(2):235–43. 28700251 10.1080/09273948.2017.1332231

[14] Kawano H, Sonoda S, Yamashita T, Maruko I, Iida T, Sakamoto T. Relative changes in luminal and stromal areas of choroid determined by binarization of EDI-OCT images in eyes with Vogt-Koyanagi-Harada disease after treatment. Graefes Arch Clin Exp Ophthalmol Albrecht Von Graefes Arch Klin Exp Ophthalmol. 2016;254(3):421–6. 10.1007/s00417-016-3283-4 26847039

[15] Liu S, Du L, Zhou Q, Zhang Q, Hu K, Qi J, et al. The choroidal vascularity index decreases and choroidal thickness increases in Vogt-Koyanagi-Harada disease patients during a recurrent anterior uveitis attack. Ocul Immunol Inflamm. 2018;26(8):1237–43. 28914578 10.1080/09273948.2017.1343357

[16] Zhang W, Kaser-Eichberger A, Fan W, Platzl C, Schrödl F, Heindl LM. The structure and function of the human choroid. Ann Anat Anat Anz Off Organ Anat Ges. 2024 June;254:152239. 10.1016/j.aanat.2024.152239 38432349

[17] Vupparaboina KK, Nizampatnam S, Chhablani J, Richhariya A, Jana S. Automated Estimation of choroidal thickness distribution and volume based on OCT images of posterior visual section. Comput Med Imaging Graph Off J Comput Med Imaging Soc. 2015;46:315–27. 10.1016/j.compmedimag.2015.09.008 26526231

[18] Gujar R, Cagini C, Fruttini D, Corbucci R, Rasheed MA, Vupparaboina KK et al. Choroidal vascularity profile in diabetic eyes using wide field optical coherence tomography. Eur J Ophthalmol. 2022;11206721221143161. 10.1177/11206721221143161 36457221

[19] Jacob N, Tyagi M, Chhablani J, Narayanan R, Kelgaonkar A, Jain M, et al. Retinal pigment epithelial characteristics in acute and resolved Vogt-Koyanagi-Harada disease. J Clin Med. 2023;12(6):2368. 36983367 10.3390/jcm12062368 PMC10054856

[20] Ganesh SK, Mistry S, Nair N. Role of swept source optical coherence tomography in management of acute Vogt-Koyanagi-Harada’s disease. Indian J Ophthalmol. 2022 July;70(7):2458–63. 10.4103/ijo.IJO_1944_21 PMC9426143 35791133

[21] Maruko I, Iida T, Sugano Y, Oyamada H, Sekiryu T, Fujiwara T, et al. Subfoveal choroidal thickness after treatment of Vogt-Koyanagi-Harada disease. Retina Phila Pa. 2011;31(3):510–7. 10.1097/IAE.0b013e3181eef053 20948460

[22] Uppugunduri SR, Rasheed MA, Richhariya A, Jana S, Chhablani J, Vupparaboina KK. Automated quantification of haller’s layer in choroid using swept-source optical coherence tomography. PLoS ONE. 2018;13(3):e0193324. 29513735 10.1371/journal.pone.0193324 PMC5841756

[23] Nkrumah G, Maltsev DS, Manuel PEA, Rasheed MA, Cozzi M, Ivernizzi A, et al. Current choroidal imaging findings in central serous chorioretinopathy. Vis Basel Switz. 2020;4(4):44. 10.3390/vision4040044 PMC7712239 33081096

[24] Hattenbach LO, Falk B, Nürnberger F, Koch FHJ, Ohrloff C. Detection of inducible nitric oxide synthase and vascular endothelial growth factor in choroidal neovascular membranes. Ophthalmol J Int Ophtalmol Int J Ophthalmol Z Augenheilkd. 2002;216(3):209–14. 10.1159/000059634 12065859

[25] Ando A, Yang A, Mori K, Yamada H, Yamada E, Takahashi K, et al. Nitric oxide is proangiogenic in the retina and choroid. J Cell Physiol. 2002;191(1):116–24. 11920687 10.1002/jcp.10083

[26] Hirose S, Saito W, Yoshida K, Saito M, Dong Z, Namba K, et al. Elevated choroidal blood flow velocity during systemic corticosteroid therapy in Vogt-Koyanagi-Harada disease. Acta Ophthalmol (Copenh). 2008;86(8):902–7. 10.1111/j.1755-3768.2008.01384.x 19016661

[27] Hirooka K, Saito W, Namba K, Takemoto Y, Mizuuchi K, Uno T, et al. Relationship between choroidal blood flow velocity and choroidal thickness during systemic corticosteroid therapy for Vogt-Koyanagi-Harada disease. Graefes Arch Clin Exp Ophthalmol Albrecht Von Graefes Arch Klin Exp Ophthalmol. 2015;253(4):609–17. 10.1007/s00417-014-2927-5 25619665

[28] Mawatari Y, Hirata A, Fukushima M, Tanihara H. Choroidal dye filling velocity in patients with Vogt-Koyanagi-Harada disease. Graefes Arch Clin Exp Ophthalmol Albrecht Von Graefes Arch Klin Exp Ophthalmol. 2006;244(8):1056–9. 10.1007/s00417-005-0238-6 16411096

